# Idiopathic interstitial pneumonias: review of the latest American Thoracic Society/European Respiratory Society classification

**DOI:** 10.1590/0100-3984.2016.0134

**Published:** 2018

**Authors:** Daniel Simões Oliveira, José de Arimatéia Araújo Filho, Antonio Fernando Lins Paiva, Eduardo Seigo Ikari, Rodrigo Caruso Chate, César Higa Nomura

**Affiliations:** 1 Instituto do Coração do Hospital das Clínicas da Faculdade de Medicina da Universidade de São Paulo (InCor/HC-FMUSP), São Paulo, SP, Brazil.

**Keywords:** Idiopathic interstitial pneumonia, American Thoracic Society, European Respiratory Society, Pneumonias intersticiais idiopáticas, American Thoracic Society, European Respiratory Society

## Abstract

The diagnosis of idiopathic interstitial pneumonias (IIPs) involves a multidisciplinary scenario in which the radiologist assumes a key role. The latest (2013) update of the IIP classification by the American Thoracic Society/European Respiratory Society proposed some important changes to the original classification of 2002. The novelties include the addition of a new disease (idiopathic pleuroparenchymal fibroelastosis) and the subdivision of the IIPs into four main groups: chronic fibrosing IIPs (idiopathic pulmonary fibrosis and nonspecific interstitial pneumonia); smoking-related IIPs (desquamative interstitial pneumonia and respiratory bronchiolitis-associated interstitial lung disease); acute or subacute IIPs (cryptogenic organizing pneumonia and acute interstitial pneumonia); rare IIPs (lymphoid interstitial pneumonia and idiopathic pleuroparenchymal fibroelastosis); and the so-called “unclassifiable” IIPs. In this study, we review the main clinical, tomographic, and pathological characteristics of each IIP.

## Abstract (pt)

Resumo

O diagnóstico das pneumonias intersticiais idiopáticas (PIIs) envolve um cenário multidisciplinar no qual o radiologista assume papel fundamental. A última atualização (2013) da classificação das PIIs pela American Thoracic Society/European Respiratory Society propõe algumas mudanças importantes em relação à classificação original de 2002. Dentre as novidades, destacam-se o acréscimo de uma nova doença (fibroelastose pleuroparenquimatosa idiopática) e a subdivisão das PIIs em quatro grupos principais: PIIs crônicas fibrosantes (fibrose pulmonar idiopática e pneumonia intersticial não específica); PIIs relacionadas ao tabagismo (pneumonia intersticial descamativa e bronquiolite respiratória com doença intersticial pulmonar); PIIs agudas/subagudas (pneumonia em organização e pneumonia intersticial aguda); PIIs raras (pneumonia intersticial linfocítica e fibroelastose pleuroparenquimatosa idiopática); além das ditas “inclassificáveis”. Foram revisadas, de forma didática neste estudo, as principais características clínicas, tomográficas e patológicas de cada uma das PIIs.

## INTRODUCTION

Idiopathic interstitial pneumonias (IIPs) are part of a broad, heterogeneous group of interstitial lung diseases that encompasses more than 200 acute or chronic diseases with varying degrees of inflammation or fibrosis. Given the innumerable clinical, radiological, and pathological aspects, as well as the constant need to analyze the course of IIPs, it is essential that the diagnosis and follow-up of these diseases be conducted within a multidisciplinary scenario in which the radiologist assumes a fundamental role. High-resolution computed tomography of the chest has been the subject of recent publications in the radiology literature of Brazil^(1-7)^. The integrated approach notwithstanding, the final classification and definitive diagnosis might not be achieved in all cases. Some cases do not meet the criteria for classification into any of the classic interstitial lung disease categories and are therefore designated “unclassifiable”. Such cases continue to pose a challenge for the entire multidisciplinary team.

The most recent (2013) update of the American Thoracic Society/European Respiratory Society IIP classification^(8,9)^ proposes some important changes in relation to the original (2002) classification. Notable among those changes are the subdivision of IIPs into four main groups (chronic fibrosing, smoking-related, acute/subacute, and rare) and the addition of a new disease: idiopathic pleuroparenchymal fibroelastosis.

In this study, we provide a brief clinical description of each of these four IIP groups, as well as presenting their main radiological characteristics (Table 1), including tomographic findings and distribution patterns, together with the main differential diagnoses. The images presented were selected from the teaching files of our institution, from cases in which the tomographic pattern was typical of each IIP, with pathological confirmation.

**Table 1:** Pattern of distribution, tomographic findings, and main differential diagnoses of each IIP.

IIP category	Pattern of distribution	Tomographic findings	Differential diagnoses
Chronic fibrosis			
Idiopathic pulmonary fibrosis Nonspecific interstitial pneumonia	Peripheral, subpleural, basal Peripheral, basal, symmetric	Reticular opacities; honeycombing; minimal ground-glass opacity; architectural distortion Extensive ground-glass opacity; irregular linear opacities; traction bronchiectasis; sub-pleural preservation	Collagen diseases; hypersensitivity pneumo-nia; pneumoconiosis Usual interstitial pneumonia; desquamative interstitial pneumonia; cryptogenic organizing pneumonia; hypersensitivity pneumonia
Smoking related			
Desquamative interstitial pneumonia Respiratory bronchiolitis-associated interstitial lung disease	Lower fields, predominantly peripheral Principally upper fields, centrilobular	Ground-glass attenuation; cysts; reticular opacities Bronchial wall thickening; centrilobular nodules; ground-glass opacities	Respiratory bronchiolitis-associated interstitial lung disease; nonspecific interstitial pneumonia; hypersensitivity pneumonia Desquamative interstitial pneumonia; non-specific interstitial pneumonia; hypersensitivity pneumonia
Acute/subacute			
Cryptogenic organizing pneumonia Acute interstitial pneumonia	Subpleural, peribronchial Diffuse or focal	Focal ground-glass opacities; consolidations; reversed halo sign Consolidations; ground-glass opacities; traction bronchiectasis	Infection; aspiration; eosinophilic pneumonia; vasculitis Hydrostatic edema; acute respiratory distress syndrome; alveolar hemorrhage
Rare			
Lymphocytic interstitial pneumonia Idiopathic pleuroparenchymal fibroelastosis	Predominantly in the upper lung fields Peripheral, upper fields	Thin-walled cysts; centrilobular nodules; ground-glass attenuation; peribronchovascular septal thickening Pleural thickening; subpleural fibrotic changes	Nonspecific interstitial pneumonia; sarcoidosis; histiocytosis X; cystic lung diseases Pneumoconiosis; hypersensitivity pneumo-nia; familial pulmonary fibrosis

## CHRONIC FIBROSING IIPs

### Idiopathic pulmonary fibrosis (Figure 1)

**Figure 1:**
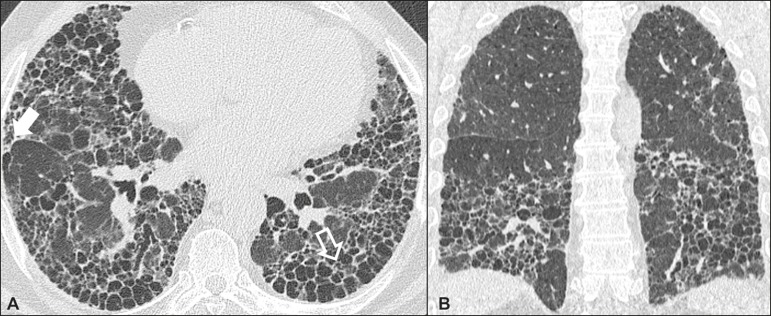
Idiopathic pulmonary fibrosis. A 66-year-old male smoker. Axial high-resolution computed tomography scan of the chest (**A**) and coronal reformatting (**B**). In **A**, fine reticular opacities (closed arrow), with traction bronchiectasis, traction bronchiolectasis, and honeycombing (open arrow). In **B**, predominantly peripheral and basal pattern of distribution.

Among all IIPs^(10)^, idiopathic pulmonary fibrosis is the most common; it is an interstitial lung disease of unknown cause, characterized histologically by the usual interstitial pneumonia pattern^(11,12)^, with dispersed fibroblastic foci and heterogeneous involvement of the parenchyma, areas of tissue preservation being interspersed with areas of interstitial inflammation and honeycombing. It occurs mainly in adults over 50 years of age who are smokers or former smokers, typically manifesting as progressive dyspnea and dry cough. In general, it has a poor prognosis, with an estimated survival of less than five years after the diagnosis. Patients usually have fewer acute exacerbations when treated with cyclosporine combined with corticosteroids, and most are considered candidates for lung transplantation^(10)^. In an appropriate clinical setting (typical clinical and radiological findings), the diagnosis of idiopathic pulmonary fibrosis can be established without the need for biopsy^(10)^.

### Nonspecific interstitial pneumonia (Figure 2)

**Figure 2:**
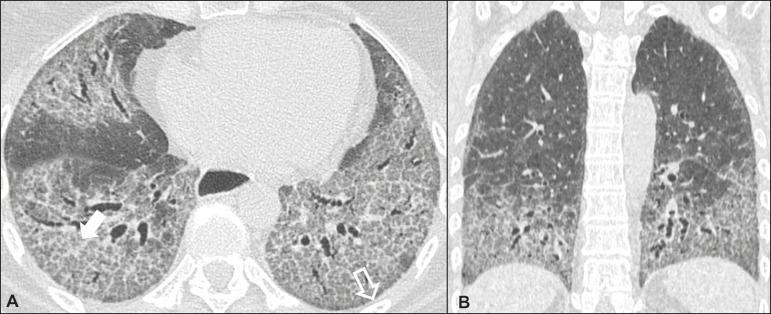
Nonspecific interstitial pneumonia. A 51-year-old female patient with scleroderma. Axial high-resolution computed tomography scan of the chest (**A**) and coronal reformatting (**B**). In **A**, ground-glass attenuation, with linear reticular opacities (closed arrow), traction bronchiectasis, and traction bronchiolectasis. Note the discrete subpleural preservation (open arrow). In **B**, predominantly basal and symmetric pattern of distribution.

Nonspecific interstitial pneumonia is characterized histologically by homogeneous inflammation and expansion of the alveolar walls, with or without fibrosis; it can be classified as belonging to one of three subtypes^(11)^: cellular (less common and with a better prognosis); fibrotic (with a worse prognosis); or mixed. Nonspecific interstitial pneumonia can be idiopathic, although it most often manifests as pulmonary symptoms associated with connective tissue diseases (especially scleroderma), hypersensitivity pneumonia, drug reactions, or diffuse alveolar damage. It typically occurs in women between 40 and 50 years of age, with symptoms similar to, although usually milder than, those of idiopathic pulmonary fibrosis. Treatment is directed to the underlying disease and can include the use of a combination of systemic corticosteroids and cytotoxic drugs, which is successful in most cases^(10)^. Although the tomographic findings of idiopathic pulmonary fibrosis and nonspecific interstitial pneumonia are often similar^(13)^, an experienced radiologist knows the main differences between them (Table 1).

## SMOKING-RELATED IIPs

### Desquamative interstitial pneumonia (Figure 3)

**Figure 3:**
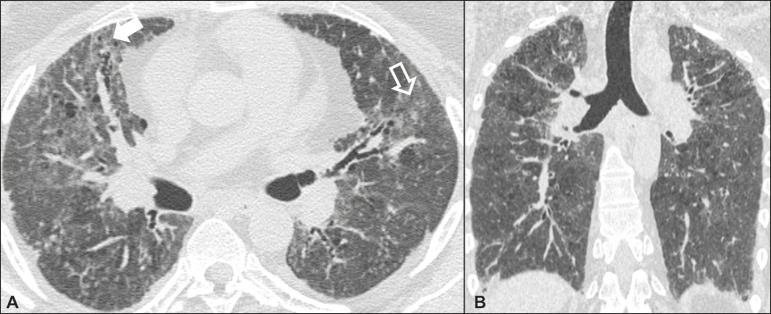
Desquamative interstitial pneumonia. A 65-year-old male smoker. Axial high-resolution computed tomography scan of the chest (**A**) and coronal reformatting (**B**). In **A**, linear reticular opacities (closed arrow), with sparse bilateral ground-glass opacity (open arrow). In **B**, predominantly peripheral pattern of distribution.

Desquamative interstitial pneumonia is a rare disease that is strongly associated with smoking; in some cases, it progresses to respiratory failure and advanced pulmonary fibrosis^(8)^. Histopathological findings include homogeneous thickening of the alveolar septa and intra-alveolar accumulation of pigmented macrophages^(11)^. Men between 30 and 50 years of age constitute the majority of patients; after smoking cessation and corticosteroid therapy, there is significant improvement of the symptoms^(11)^.

### Respiratory bronchiolitis-associated interstitial lung disease (Figure 4)

**Figure 4:**
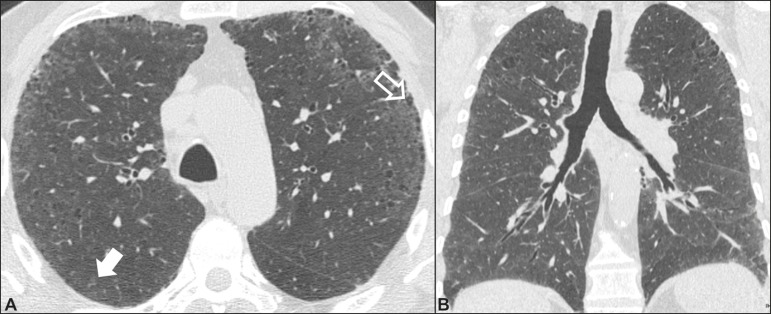
Respiratory bronchiolitis-associated interstitial lung disease. A 53-year-old male smoker. Axial high-resolution computed tomography scan of the chest (**A**) and coronal reformatting (**B**). In **A**, diffuse centrilobular opacities (closed arrow), thickening of the bronchial walls and paraseptal/centrilobular emphysema (open arrow). In **B**, distribution predominantly in the upper fields.

Classically characterized by the combination of interstitial disease and respiratory bronchiolitis, the typical histological findings of respiratory bronchiolitis-associated interstitial lung disease include the accumulation of pigmented macrophages in the respiratory bronchioles, alveolar ducts, and adjacent alveoli, together with minimal fibrosis and inflammatory infiltrate^(11)^. The affected patients are usually men between 30 and 50 years of age who have few or no symptoms. The treatment consists of smoking cessation and corticosteroid therapy^(10)^.

## ACUTE AND SUBACUTE IIPs

### Cryptogenic organizing pneumonia (Figure 5)

**Figure 5:**
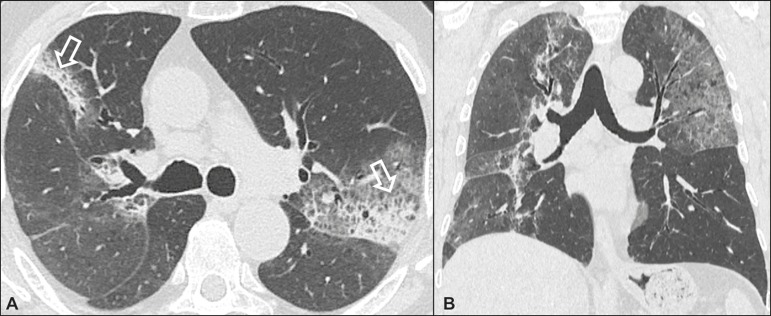
Cryptogenic organizing pneumonia. A 62-year-old female patient with a one-year history of dyspnea and a two-month history of symptom worsening. Axial high-resolution computed tomography scan of the chest (**A**) and coronal reformatting (**B**). In **A**, ground-glass opacities/sparse bilateral consolidations (open arrows). In **B**, bilateral subpleural and peribronchial pattern of distribution.

Formerly known as bronchiolitis obliterans organizing pneumonia, organizing pneumonia is characterized histologically by buds of granulation tissue within the alveolar ducts and adjacent alveoli, accompanied by chronic inflammatory infiltrate involving the interstitium and alveolar spaces^(8)^. That is a common pattern of response to various types of lung injury and is most commonly found in conjunction with connective tissue diseases, drug-induced adverse pulmonary reactions, hypersensitivity pneumonia, infectious processes, and aspiration, among others. When no cause is identified, it is known as cryptogenic organization pneumonia^(10)^. It predominantly affects individuals between 50 and 70 years of age, with no gender preference; most patients seek treatment in one to three months after the symptom onset, which is often preceded by respiratory tract infections^(11)^. Corticosteroid treatment usually produces an excellent response^(9)^.

### Acute interstitial pneumonia (Figure 6)

**Figure 6:**
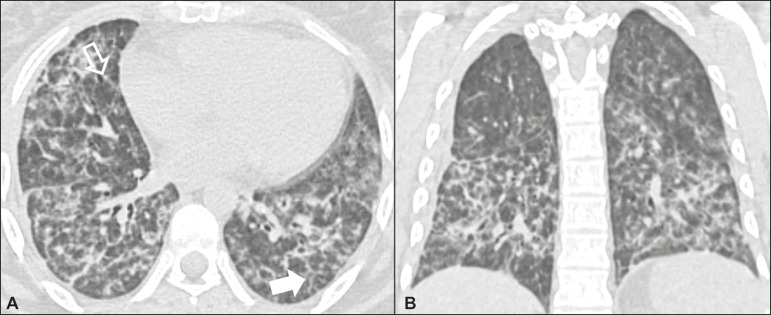
Acute interstitial pneumonia. A 32-year-old female patient, hospitalized for severe dyspnea two weeks prior. Axial high-resolution computed tomography scan of the chest (**A**) and coronal reformatting (**B**). In **A**, diffuse ground-glass attenuation pattern, with some traction bronchiectasis (open arrow) and reticular opacities (closed arrow). In **B**, bilateral diffuse, symmetrical pattern of distribution.

Acute interstitial pneumonia is an extremely severe idiopathic acute interstitial disease, characterized by a histopathological pattern of diffuse alveolar damage, the exudative phase of which is defined by interstitial and intra-alveolar edema, formation of hyaline membranes, and diffuse alveolar infiltration of inflammatory cells. It has a poor prognosis (mortality greater than 50%), mainly affecting previously healthy patients between 50 and 60 years of age, with severe sudden-onset dyspnea (occurring within the first three weeks), typically progressing to the need for mechanical ventilation^(10)^. The clinical, radiological, and histological findings are the same as those of acute respiratory distress syndrome^(11)^. The treatment is basically supportive, with oxygen supplementation, and the use of corticosteroids is effective in the acute phase^(10)^.

## RARE IIPs

### Lymphocytic interstitial pneumonia (Figure 7)

**Figure 7:**
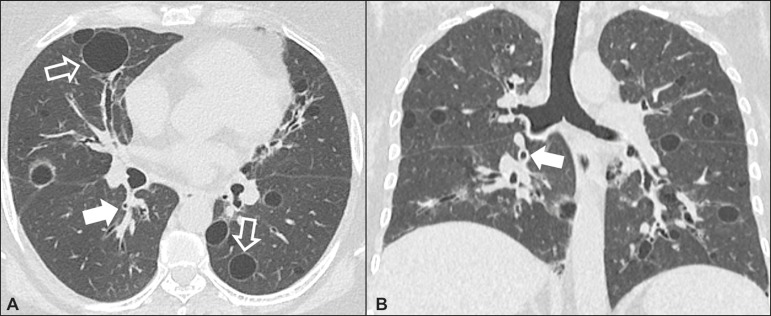
Lymphocytic interstitial pneumonia. A 62-year-old female patient with Sjögren’s syndrome. Axial high-resolution computed tomography scan of the chest (**A**) and coronal reformatting (**B**). In **A**, diffuse thickening of the bronchial walls (closed arrows), some ground-glass opacities and thin-walled cysts of varying sizes, with a diffuse, bilateral distribution (open arrows). In **B**, distribution predominantly in the lower fields.

In the vast majority of patients, lymphocytic interstitial pneumonia is associated with systemic autoimmune or immunodeficiency disorders, including connective tissue diseases (mainly Sjögren's syndrome, autoimmune thyroiditis, and primary biliary cirrhosis). In its idiopathic form, it is considered extremely rare. Histologically, it is characterized by diffuse infiltration of the interstitium by polyclonal lymphocytes, histiocytes, and variable numbers of plasma cells, as well as reactive lymphoid follicles distributed throughout the peribronchovascular regions, accompanied by intense inflammation^(11)^. It mainly affects women between 50 and 70 years of age, with nonspecific symptoms of chronic coughing and dyspnea (typically for more than three years). The response to corticosteroid therapy is unpredictable^(10)^.

### Idiopathic pleuroparenchymal fibroelastosis (Figure 8)

**Figure 8:**
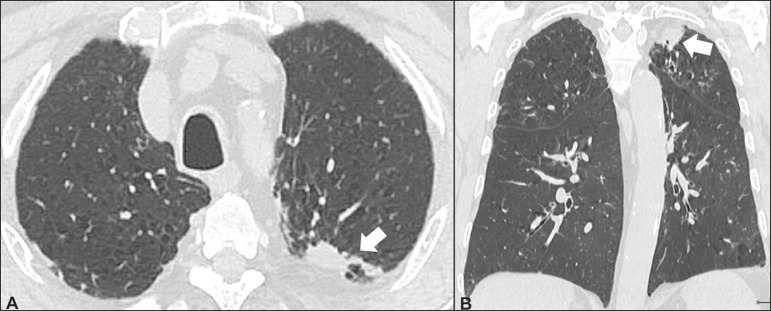
Idiopathic pleuroparenchymal fibroelastosis. An 80-year-old male smoker with a one-year history of dyspnea on minimal exertion. Axial high-resolution computed tomography scan of the chest (**A**) and coronal reformatting (**B**). In **A**, pleural thickening with subpleural fibrotic changes (closed arrows). In **B**, asymmetric peripheral pattern of distribution in the upper fields.

Idiopathic pleuroparenchymal fibroelastosis is a rare disease affecting the pleura and lungs. It is characterized histologically by homogeneous subpleural fibrosis and abundant elastic fibers, with variable inflammation and lymphoid aggregates. Currently, there is no consensus regarding its diagnostic criteria or regarding the fact that it is a new entity^(14)^. With a little more than 40 cases described in the literature to date, this entity has been associated with infections, bone marrow transplantation, autoimmune diseases, and genetic predisposition. Its symptoms are nonspecific (dry cough and dyspnea), and it is believed to progress slowly (over the course of 10-20 years), with few therapeutic options other than lung transplantation^(15)^.

## CONCLUSION

The correct diagnosis and classification of IIPs involves a broad multidisciplinary debate, with many gaps. Given the similarity of the clinical and pathological findings, together with the complexity of the differential diagnoses and the need for constant follow-up, high-resolution computed tomography plays a fundamental role in the diagnostic assessment of these diseases.
